# Association between diabetes mellitus and poor patient outcomes after out-of-hospital cardiac arrest: A systematic review and meta-analysis

**DOI:** 10.1038/s41598-018-36288-1

**Published:** 2018-12-18

**Authors:** Dinesh Chandra Voruganti, Adithya Chennamadhavuni, Rohan Garje, Ghanshyam Palamaner Subash Shantha, Marin L. Schweizer, Saket Girotra, Michael Giudici

**Affiliations:** 1 Department of Internal Medicine, University of Iowa Hospitals and Clinics, Iowa City, Iowa USA; 2 Division of Hematology and Oncology, University of Iowa Hospitals and Clinics, Iowa City, Iowa USA; 3 University of Iowa Carver College of Medicine, Iowa City, Iowa USA; 4 Division of Cardiology, University of Iowa Hospitals and Clinics, Iowa City, USA

## Abstract

Diabetes mellitus (DM) serves as an important prognostic indicator in patients with cardiac-related illness. Our objective is to compare survival and neurological outcomes among diabetic and non-diabetic patients who were admitted to the hospital after an out-of-hospital cardiac arrest (OHCA). We searched MEDLINE and EMBASE for relevant articles from database inception to July 2018 without any language restriction. Studies were included if they evaluated patients who presented with OHCA, included mortality and neurological outcome data separately for DM patients and Non-DM patients and reported crude data, odds ratio (OR), relative risk (RR) or hazard ratio (HR). Two investigators independently reviewed the retrieved citations and assessed eligibility. The quality of included studies was evaluated using Newcastle-Ottawa quality assessment scale for cohort studies. Random-effect models using the generic variance method were used to create pooled odds ratios (OR) and 95% confidence intervals (CI). Heterogeneity was assessed using the *I*^2^ value. Survival and neurological outcomes (using modified rankin scale and cerebral performance category scale) after OHCA in hospitalized patients with DM compared with patients without DM. Out of 57 studies identified, six cohort studies met the inclusion criteria. In an analysis of unadjusted data, patients with DM had lower odds of survival, pooled OR 0.64; 95% CI, 0.52–0.78, [I^2^ = 90%]. When adjusted ORs were pooled, the association between DM and survival after OHCA was still significantly reduced, pooled OR 0.78, 95% CI, 0.68–0.89 [I^2^ = 55%]. Unadjusted pooled OR revealed poor neurological outcomes in patients with DM, pooled OR 0.55, 95% CI, 0.38–0.80 [I^2^ = 90%]. The result demonstrates significant poor outcomes of in-hospital survival and neurological outcomes among DM patients after OHCA.

## Introduction

Sudden cardiac arrest (SCA) is reported to account for approximately 15 percent of the total mortality in the United States and other industrialized countries^1^. In 1999, the estimated number of sudden cardiac deaths in the United States was approximately 450,000^2^. Recent studies had demonstrated improved survival and neurological outcomes after cardiac arrest^3^. However, the opportunity to improve the outcomes after cardiac arrest remains to be explored. Most of the risk factors for coronary heart disease (CHD) are also risk factors for SCA, out of which, diabetes mellitus (DM) is considered one of the most important risk factors. Interestingly, type 2 DM patients have been reported to have a 2 to a 4-fold increased risk of out-of-hospital cardiac arrest (OHCA) compared with nondiabetic patients^4^. Moreover, in an observational study on OHCA, there was a statistically significant reduction in the probability of survival to hospital discharge among patients with chronic conditions, such as congestive heart failure, prior myocardial infarction, hypertension, and diabetes (odds ratio (OR) 0.84 for each additional chronic condition)^5^. Multiple observational studies have demonstrated an association between DM and decreased survival after OHCA. However, the studies have had varying results. Therefore, to further investigate this relationship, we conducted a systematic literature review and meta-analysis with an intention to summarize all published clinical evidence.

## Methods

This study was conducted per PRISMA (Preferred Reporting Items for Systematic Reviews and Meta-Analysis) statement (Supplementary Table). A systematic literature search of MEDLINE and EMBASE was carried out from inception to July 2018 to identify original studies that investigated the association between DM and survival after OHCA. The systematic literature review was independently conducted by two investigators (DV, AC) using a search strategy that included terms for “diabetes mellitus” and “out-of-hospital cardiac arrest” in human subjects as described in the supplementary table. No language limitation was applied. The last search was performed on July 4, 2018. We contacted the study authors for additional information. Literature search details are explained in the supplementary table. A manual search for further potentially relevant studies using references of the included articles was also performed.

Inclusion criteria also included the following:

1. Observational studies (cross-sectional, case-control or cohort studies) and randomized controlled trials that involved diabetic patients who presented to the hospital after surviving an OHCA.
2. Studies that reported the survival after hospitalization. Hospital survival outcomes were chosen as study outcomes because, though arbitrary, in-hospital mortality will represent a reliable time point to assess short-term mortality where cardiovascular risk factors will play a predominant role. We excluded case reports and case series.

The same investigators independently reviewed the retrieved articles for their eligibility. The Newcastle-Ottawa quality assessment scale (NOS) was used to appraise the quality of included studies in 3 areas including study selection, study comparison, and determination of the outcome of interest^6^.

Two co-authors (DV and AC) independently extracted data from the included full-text citations. A structured data collection form was used to extract the following data from each study: title of this study, name of the first author, publication year, study dates, country where this study was conducted, number of subjects, demographic data, definition of DM, methods used to identify and verify DM, OHCA, adjusted effect estimates (odds ratio) with 95% CI and covariates that were adjusted in the multivariable analysis. A third investigator (RG) reviewed this data extraction process to ensure accuracy. All disagreements were resolved with discussion between the two abstractors. Data analysis was performed using the REVIEW MANAGER 5.3 software from the Cochrane Collaboration (London, UK). Unadjusted point estimates from each study were pooled using the inverse-variance method. Adjusted point estimates from each study were pooled using the inverse-variance weighting as described by DerSimonian and Laird, which assigned the weight of each study based on its variance^7^. In light of the high likelihood of heterogeneity due to different study designs, populations and definition of DM, random-effect models were used. Cochran’s Q test and the I^2^ statistic were used to determine the between-study heterogeneity. A value of *I*^2^ of 0–25% represents insignificant heterogeneity, 26–50% represents low heterogeneity, 51–75% represents moderate heterogeneity, and more than 75% represents high heterogeneity^8^. For all analyses, a *P*-value of <0.05 was considered statistically significant. A subgroup analysis was performed to assess the study heterogeneity by including studies which have performed robust adjustment for comorbidities (such as chronic obstructive pulmonary disease (COPD)). Sensitivity analysis was also performed to test the robustness of the output and quantify the model’s uncertainty by excluding each study one at a time. Funnel plot analysis was performed to evaluate for publication bias.

## Results

**Figure 1:**
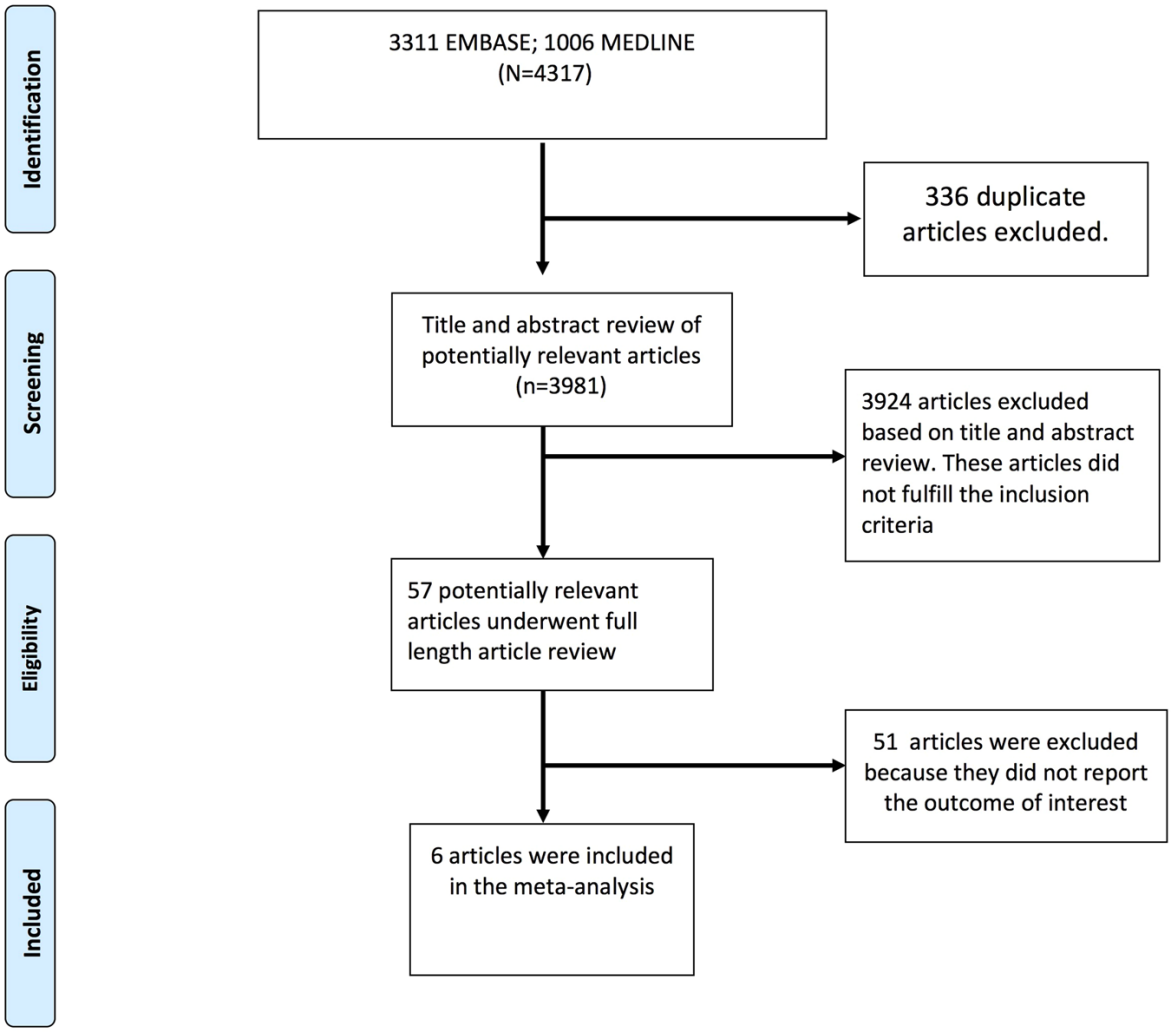
CONSORT Diagram. The figure outlines our search methodology. A total of 6 studies met our study inclusion criteria and were included in the meta-analysis.

From a total of 4317 citations identified, 57 studies^9–68^ underwent full-length review. Of these, only 6 cohort studies^47,52,57,61,66,68^ were included for meta-analysis. Figure 1 delineates the study selection details. All the six included studies were performed in different parts of the world, including 2 Asian studies, 2 European studies, a study from Canada and a study from Australia. All studies are cohort studies (5 retrospective and one prospective cohort study). All the studies included participants based on the utstein guidelines. Most studies adjusted for age and gender. The 6 included studies involved 41,561 patients: 10,871 with DM and 30,690 without DM. Table 1 summarizes the included studies in our analysis.

**Table 1:** Characteristics of Studies included in the Analysis.

Study (year)	Van Hoeijen (2015)	Ro, Y S (2015)	Parry M (2017)	Nehme (2016)	Larsson, M (2005)	Jang DB (2015)
Country	Netherlands	South Korea	Ontario	Victoria, Australia	Sweden	Seoul, republic of Korea.
Study Design	Prospective Cohort study	Retrospective Cohort study	Retrospective Cohort Study	Retrospective Cohort study	Retrospective Cohort study	Retrospective Cohort study
Year of publication	2015	2015	2017	2016	2005	2015
Number of participants	DM: 134; Non-DM: 711	DM 2639; Non-DM 7096	DM 2807; Non-DM 7279	DM 2438; Non-DM: 9435	DM 187; Non-DM 1190	DM 2651; Non-DM 4932
Participants	Data from ARREST (AmsteRdam REsuscitation STudies) registry of OHCA in the Netherlands, the study period June 2005 to January 2010.	Korean national OHCA database composed of hospital and ambulance data.	Population-based registry of consecutive OHCA Ontario.	Retrospective analysis of data from a statewide cardiac arrest registry in Victoria, Australia	Data on the entire cardiac arrest cohort were obtained from the Goteborg emergency medical service.	Emergency Medical services (EMS)-assessed OHCA cases with presumed cardiac etiology survived to admission in South Korea during the period of January 1, 2009, to December 31, 2013,
Mean age of participants (in years)	DM: 70.1; Non-DM: 64.4	DM 68; Non-DM 59	DM 72; Non-DM 69	DM 72; non-DM 69 (median age)	DM 70; Non-DM 66	DM 68; non-DM 57 (median age)
Diagnosis of diabetes	Type-2 diabetes mellitus defined as the prescription of at least oneglucose-lowering drug (ATC A10) within six months before OHCA. These patients were stratified into three categories according to medication prescriptions at OHCA date: (i) oral glucose-lowering drugs (OGLDs) only; (ii) at least one OGLD plus insulin: (iii) insulin only	DM variable was obtained from medical record review. DM was positive when the patient had a clinical history diagnosed by aa physician before the arrest event.	Diabetes status was ascertained using a variable based on the in-hospital record.	DM variable was obtained from medical record review.	Data obtained from hospital records and general practitioner records.	Diabetes mellitus was recorded positive when the patient had a clinical history of diabetes diagnosis by a physician before the arrest
Cardiac arrest cohort	OHCA with documented VT/VF (ventricular tachycardia/Ventricular fibrillation) and a clear non-cardiac cause was absent.	All adults who are older than 18 years and survived to admission with presumed cardiac etiology were included.	Adults ≥18years of age who experienced an OHCA, had data on diabetes status, and were treated by EMS between 2012–2014 were included in the analysis.	Patients aged over 15 years who experienced an OHCA of presumed cardiac etiology and received an attempted resuscitation by emergency medical services (EMS) between 1 January 2007 and 30 June 2015. Patients who were witnessed to arrest by EMS personnel were excluded.	Patients suffering an out-of-hospital cardiac arrest between 1 October 1980 and 1 October 2003 were included in the survey, regardless of the cause of the arrest and age.	Cases were presumed to be of cardiac etiology if there were no definite sites of non-cardiac etiology such as evident trauma, asphyxia or hanging, drowning, poisoning, and burn.
Confounder adjustment	age, sex, pre-existing cardiovascular diseases (CVD), acute myocardial infarction (MI), obstructive pulmonary disease, and resuscitation parameters (OHCA at a public location, by- stander witnessed OHCA, bystander cardiopulmonary resuscitation performed, use of an automated external defibrillator (AED), time between emergency call and EMS arrival)	Hypothermia, diabetes mellitus, age, gender, hypertension, heart disease, stroke, primary electrocardiogram, community, arrest place, witness, bystander CPR, prehospital defibrillation, EMS response time, EMS scene time, EMS transport time, prehospital ROSC, and emergency department (ED) level.	Diabetes diagnosis, Age in years,Male gender,Location (ref = Private/Residential) Public OtherFirst-response CPR (ref = none) Bystander EMSWitnessedObvious causeShockable first monitored rhythm	Age increase per year, Male, Pre-existing conditions Hypertension, Dyslipidemia, Heart failure, Arrhythmia, Chronic obstructive pulmonary disease, Stroke or transient ischemic attack, Diabetes, Initial shockable rhythm, Diabetes × initial shockable rhythm†, Response time increase of emergency medical services (per minute), Public location Bystander witnessed, Bystander cardiopulmonary resuscitation, Metropolitan region	Age, a history of myocardial infarction,angina pectoris, hypertension, and heart failure, chronic obstructive pulmonary disease and cardiac history.	Diabetes mellitus, cardiac disease, patient age, gender, place of arrest (private vs. public), bystander CPR performed, metropolitan, EMS response time interval, EMS scene time interval, EMS transport time interval, EMS defibrillation, ED level, reperfusion therapy, and hypothermia therapy
Quality assessment (Newcastle-Ottawa scale)	Selection -4 Comparability – 1 Outcome - 3	Selection -4 Comparability – 1 Outcome - 3	Selection -4 Comparability – 1 Outcome - 3	Selection -4 Comparability – 1 Outcome - 3	Selection -4 Comparability – 1 Outcome - 3	Selection -4 Comparability – 1 Outcome - 3

**Figure 2:**
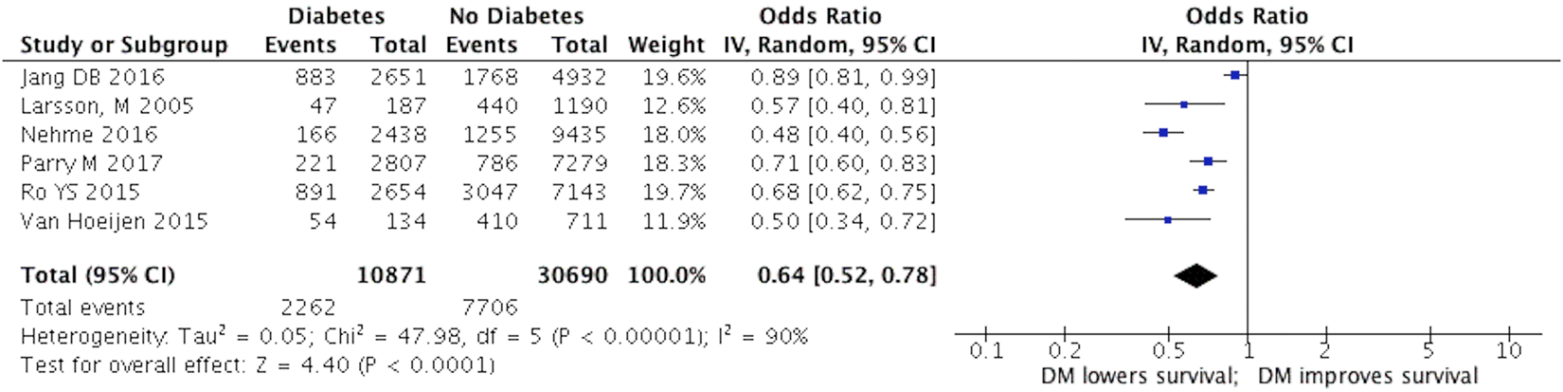
Association of diabetes with survival among patients with OHCA**:** In this analysis, unadjusted odds ratio that quantify the association between diabetes and survival in OHCA patients was pooled from each study. The pooled odds ratio (black diamond) is 0.64 (95% CI [0.52–0.78]) indicating that diabetes is associated with poor survival.

**Figure 3:**
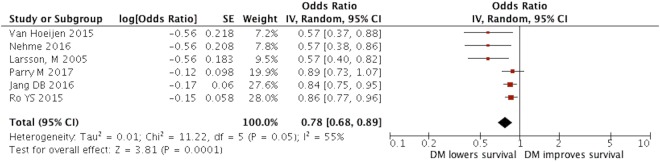
In this analysis, the adjusted odds ratio that quantifies the association between diabetes and survival in OHCA patients was pooled from each study. The pooled odds ratio (black diamond) is 0.78 (95% CI [0.68–0.89]) indicating that diabetes is associated with poor survival.

When we pooled the results of all six studies, we found a statistically significant association between the presence of DM and survival following out-of-hospital cardiac arrests with a pooled adjusted odds ratio of 0.78, 95% confidence interval (CI) 0.68–0.89, demonstrating patients with DM had poor survival following OHCA. There was moderate heterogeneity between studies with an I^2^ of 55%. Figures 2 and 3 illustrate the unadjusted and adjusted forest plot of this meta-analysis. A subgroup analysis of 3 studies which performed robust adjustment of confounders (ex: COPD) resulted in a reduced heterogeneity of I^2^ = 0%. Table 2 illustrates the sub-group analysis which demonstrates the reduction in heterogeneity. Sensitivity analysis of included studies revealed similar outcomes with minimal variability in the pooled effect estimate. Table 2 summarizes the results of the sensitivity analysis.

**Table 2:** Sensitivity Analysis and sub-group analysis for survival after hospitalization for cardiac arrest in patients with Diabetes Mellitus.

Study	Pooled odds ratio with 95% confidence interval
**All studies included (random effects)**	0.78 [0.68, 0.89]
**Study excluded (year) (Sensitivity Analysis)**	
Jang DB 2015	0.73 [0.60, 0.88]
Larrson 2005	0.82 [0.73, 0.91]
Nehme 2016	0.81 [0.72, 0.91]
Parry M 2017	0.74 [0.63, 0.87]
Ro YS 2015	0.73 [0.60, 0.88]
Van Hoeijen 2015	0.80 [0.71, 0.91]
**Sub-group analysis – Study**, **year**	**Pooled odds ratio with 95% confidence interval; Heterogeneity (I** ^**2**^ **)**
Jang DB 2016, Parry M 2017, Ro YS 2015	0.86 (0.80–0.92); I^2^ = 0%
Larrson 2005, Nehme 2016, Van Hoeijen 2015	0.57 (0.45–0.72); I^2^ = 0%

**Figure 4:**
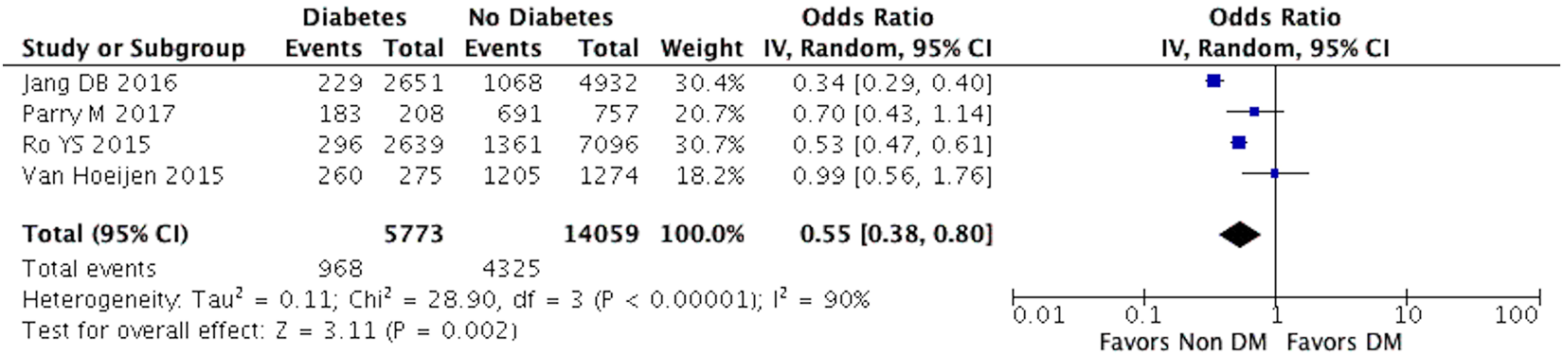
In this analysis, unadjusted odds ratio that quantifies the association between diabetes and neurological outcomes in OHCA patients was pooled from each study. The pooled odds ratio (black diamond) is 0.55 (95% CI [0.38–0.80]) indicating that diabetes is associated with poor neurological outcomes.

We also found a statistically significant association between favorable neurological outcomes in patients with DM after OHCA with a pooled unadjusted odds ratio of 0.55, 95% CI 0.38–0.80. However, there was a considerable study heterogeneity among these studies with a I^2^ of 90%. Figure 4 illustrates the forest plot of this analysis. Subgroup analysis was not performed for this outcome because the events were crude estimates, and due to a relatively small number of included studies. Sensitivity analysis revealed that there was no statistically significant pooled estimate after excluding the study by Ro YS *et al*., likely due to the exclusion of a study with a relatively large sample size influencing the outcomes. Table 3 summarizes the results of the sensitivity analysis.

**Table 3:** Sensitivity Analysis for favorable neurological outcomes after hospitalization for cardiac arrest in patients with Diabetes Mellitus.

Study	Pooled odds ratio with 95% confidence interval
All studies included (random effects model)	0.55 [0.38, 0.80]
**Studies excluded (year)**	
Jang DB 2015	0.66 [0.46, 0.95]
Parry M 2017	0.52 [0.34, 0.79]
Ro YS 2015	0.59 [0.30, 1.20]
Van Hoeijen 2015	0.48 [0.33, 0.70]

**Figure 5:**
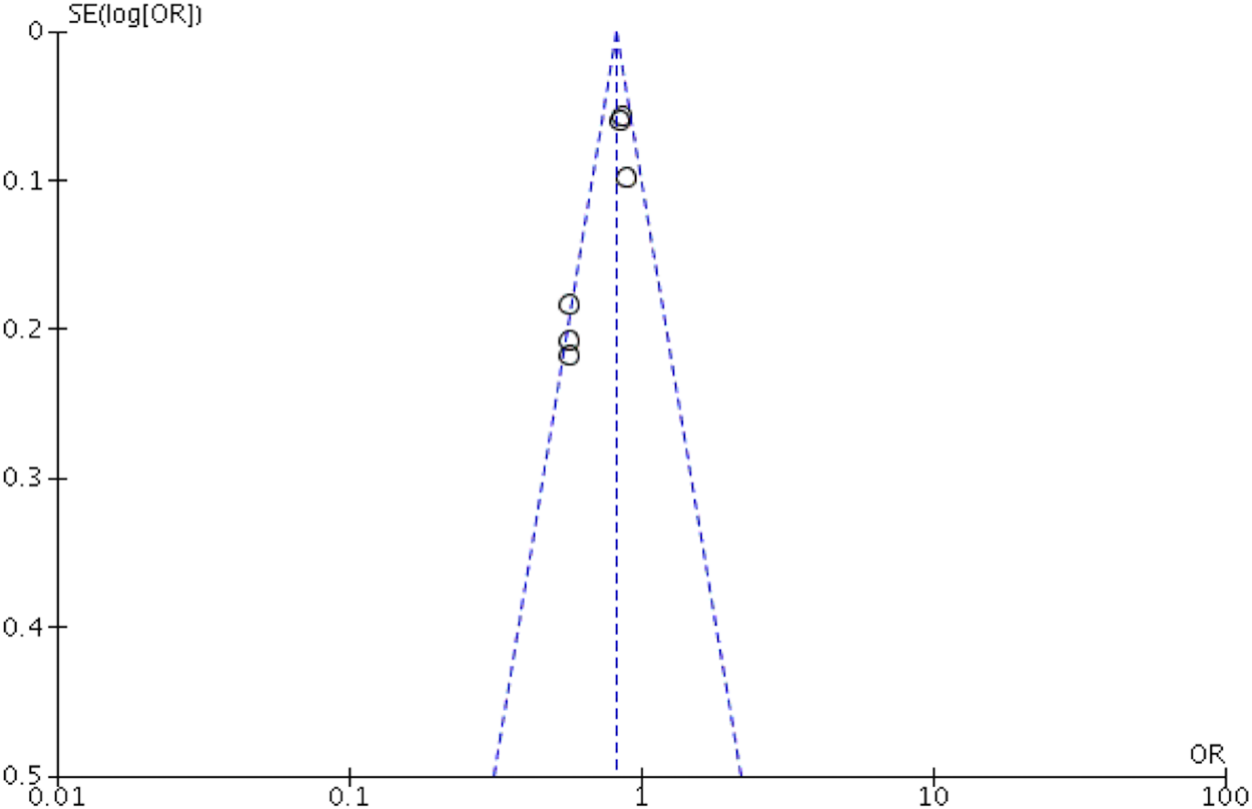
Funnel plot for adjusted odds ratio on in-hospital survival among DM and non-DM patients after OHCA reveals a low risk for publication bias given a symmetrical distribution of the included studies (black circles) clustering at the apex around the mean effect size (middle dotted line) in the funnel plot.

Figure 5 shows the funnel plot for publication bias for survival outcome after OHCA. The plot is mostly symmetric with studies mostly clustering at the apex and middle of the funnel plot. There were no studies at the bottom of the funnel plot, thus, providing a very less likelihood of publication bias.

## Discussion

This systematic review and meta-analysis compared the survival and neurological outcomes among DM and non-DM patients who were admitted to the hospital after an out-of-hospital cardiac arrest (OHCA). To our knowledge, this is the first systematic review and meta-analysis comparing outcomes after OHCA, according to diabetes. We identified a reduced survival among patients with DM with pooled adjusted OR 0.78, 95% confidence interval (CI) 0.68–0.89. A potential explanation for lower survival among DM is because patients with DM are associated with larger myocardial infarction (MI) size and reduced reperfusion leading to higher rates of morbidity and mortality. Moreover, metabolic derangements of patients with DM after OHCA (e.g., increased blood glucose variability leading to hyperglycemia or hypoglycemia) may also contribute to lower in-hospital survival rate.

Secondly, we found that patients with DM had poor neurological outcomes (n = 19,832 patients). This is statistically relevant with an unadjusted OR 0.55 (95% CI 0.38–0.80). This is likely due to pre-existing atherosclerotic vascular disease in patients with diabetes who may be at risk for a higher degree of cerebral hypoperfusion during an episode of cardiac arrest.

Although most of the included studies were of high quality as reflected by the high-quality NOS assessment scores, we acknowledge that this meta-analysis has some limitations. Majority of the included studies did not stratify the patients based on the drugs used to treat diabetes, duration of DM and the control of glucose levels by monitoring the HbA1c levels which might have influenced the survival outcomes, and might have explained the heterogeneity among the studies. Misclassification in the diagnosis of DM may have occurred, since some patients with T2DM may be treated only with nonpharmacological therapy such as lifestyle modification. The neurological outcome data in the included studies is limited due to variability of assessment tools used to quantitate neurological outcomes (Cerebral Performance Category scale in studies by Jang *et al*., Van Hoeijen *et al*. and Ro YS *et al*.; and Modified Rankin scale in the study by Parry *et al*.). Variability in the implementation of post-cardiac arrest hypothermia protocols among the four studies that evaluated neurological outcomes can also explain the heterogeneity of these results.

In summary, this meta-analysis demonstrates significantly lower odds of in-hospital survival after OHCA among DM patients compared with non-DM patients.

Considering the limitations of the data from the included studies, our results should stimulate further research with robust strategies for risk adjustment to elucidate the association between diabetes and poor cardiac outcomes. While DM is a known risk factor for SCA, with these results, future studies are required to determine whether improved blood glucose control reduces the impact of diabetes on the survival outcomes after OHCA. Because risk factors for OHCA, SCA, and CAD are modifiable, appropriate measures to optimize health care utilization in diabetic populations may reduce this gap.

## Electronic supplementary material

Supplementary Information
