# Supplementary material for: Association between diabetes mellitus and poor patient outcomes after out-of-hospital cardiac arrest: A systematic review and meta-analysis
Source: Sci Rep. 2018 Dec 18;8:17921. doi: 10.1038/s41598-018-36288-1 (PMC6298970; doi:10.1038/s41598-018-36288-1)
Supplement: Supplementary file 1 — Supplementary Information [file 41598_2018_36288_MOESM1_ESM.docx]

**Association between diabetes mellitus and poor patient outcomes after out-of-hospital cardiac arrest: A systematic review and meta-analysis.**

Dinesh Chandra Voruganti MBBS^a^;* Adithya Chennamadhavuni MBBS^a^; Rohan Garje MBBS^b^; Ghanshyam Palamaner Subash Shantha MBBS^d^; Marin L Schweizer PhD^c^ ; Saket Girotra MD^d^ ; Michael Giudici MD^d^

^a^Department of Internal Medicine, University of Iowa Hospitals and Clinics, Iowa City, Iowa, USA.

^b^Division of Hematology and Oncology, University of Iowa Hospitals and Clinics, Iowa City, Iowa, USA

^c^University of Iowa Carver College of Medicine, Iowa City, Iowa, USA

^d^Division of Cardiology, University of Iowa Hospitals and Clinics, Iowa City, USA

**Address for correspondence:**

Dinesh Chandra Voruganti, MBBS,

Department of Internal Medicine, University of Iowa Hospitals and Clinics,

200 Hawkins Dr., Internal Medicine, Iowa City, IA 52242.

Telephone number: 319-353-7858

Fax: 319-353-8073

Email: [dinesh-voruganti@uiowa.edu](mailto:dinesh-voruganti@uiowa.edu)

| Section/topic | # | Checklist item | Reported on page # |
| --- | --- | --- | --- |
| TITLE | | |  |
| Title | 1 | Identify the report as a systematic review, meta-analysis, or both. | 1 |
| ABSTRACT | | |  |
| Structured summary | 2 | Provide a structured summary including, as applicable: background; objectives; data sources; study eligibility criteria, participants, and interventions; study appraisal and synthesis methods; results; limitations; conclusions and implications of key findings; systematic review registration number. | 2 |
| INTRODUCTION | | |  |
| Rationale | 3 | Describe the rationale for the review in the context of what is already known. | 3 |
| Objectives | 4 | Provide an explicit statement of questions being addressed with reference to participants, interventions, comparisons, outcomes, and study design (PICOS). | 3,4 |
| METHODS | | |  |
| Protocol and registration | 5 | Indicate if a review protocol exists, if and where it can be accessed (e.g., Web address), and, if available, provide registration information including registration number. | 3 |
| Eligibility criteria | 6 | Specify study characteristics (e.g., PICOS, length of follow-up) and report characteristics (e.g., years considered, language, publication status) used as criteria for eligibility, giving rationale. | 4 |
| Information sources | 7 | Describe all information sources (e.g., databases with dates of coverage, contact with study authors to identify additional studies) in the search and date last searched. | 3 |
| Search | 8 | Present full electronic search strategy for at least one database, including any limits used, such that it could be repeated. | 3 |
| Study selection | 9 | State the process for selecting studies (i.e., screening, eligibility, included in systematic review, and, if applicable, included in the meta-analysis). | 4 |
| Data collection process | 10 | Describe method of data extraction from reports (e.g., piloted forms, independently, in duplicate) and any processes for obtaining and confirming data from investigators. | 4 |
| Data items | 11 | List and define all variables for which data were sought (e.g., PICOS, funding sources) and any assumptions and simplifications made. | 3,4 |
| Risk of bias in individual studies | 12 | Describe methods used for assessing risk of bias of individual studies (including specification of whether this was done at the study or outcome level), and how this information is to be used in any data synthesis. | 4 |
| Summary measures | 13 | State the principal summary measures (e.g., risk ratio, difference in means). | 3 |
| Synthesis of results | 14 | Describe the methods of handling data and combining results of studies, if done, including measures of consistency (e.g., I^2^) for each meta-analysis. | 4 |

| Section/topic | # | Checklist item | Reported on page # |
| --- | --- | --- | --- |
| Risk of bias across studies | 15 | Specify any assessment of risk of bias that may affect the cumulative evidence (e.g., publication bias, selective reporting within studies). | 4,5 |
| Additional analyses | 16 | Describe methods of additional analyses (e.g., sensitivity or subgroup analyses, meta-regression), if done, indicating which were pre-specified. | 4,5 |
| RESULTS | | |  |
| Study selection | 17 | Give numbers of studies screened, assessed for eligibility, and included in the review, with reasons for exclusions at each stage, ideally with a flow diagram. | 5 |
| Study characteristics | 18 | For each study, present characteristics for which data were extracted (e.g., study size, PICOS, follow-up period) and provide the citations. | 5 |
| Risk of bias within studies | 19 | Present data on risk of bias of each study and, if available, any outcome level assessment (see item 12). | 6 |
| Results of individual studies | 20 | For all outcomes considered (benefits or harms), present, for each study: (a) simple summary data for each intervention group (b) effect estimates and confidence intervals, ideally with a forest plot. | 5 |
| Synthesis of results | 21 | Present results of each meta-analysis done, including confidence intervals and measures of consistency. | 5 |
| Risk of bias across studies | 22 | Present results of any assessment of risk of bias across studies (see Item 15). | 6 |
| Additional analysis | 23 | Give results of additional analyses, if done (e.g., sensitivity or subgroup analyses, meta-regression [see Item 16]). | 6 |
| DISCUSSION | | |  |
| Summary of evidence | 24 | Summarize the main findings including the strength of evidence for each main outcome; consider their relevance to key groups (e.g., healthcare providers, users, and policy makers). | 6 |
| Limitations | 25 | Discuss limitations at study and outcome level (e.g., risk of bias), and at review-level (e.g., incomplete retrieval of identified research, reporting bias). | 6,7 |
| Conclusions | 26 | Provide a general interpretation of the results in the context of other evidence, and implications for future research. | 7 |
| FUNDING | | |  |
| Funding | 27 | Describe sources of funding for the systematic review and other support (e.g., supply of data); role of funders for the systematic review. | 7 |

| No. | Query | Results |  |
| --- | --- | --- | --- |
| #35 | #33 AND #34 | **3311** |  |
| #34 | #28 OR #29 OR #30 OR #31 OR #32 | **75264** |  |
| #33 | #1 OR #2 OR #3 OR #4 OR #5 OR #6 OR #7 OR #8 OR #9 OR #10  OR #11 OR #12 OR #13 OR #14 OR #15 OR #16 OR #17 OR #18 OR #19 OR #20 OR #21 OR #22  OR #23 OR #24 OR #25 OR #26 OR #27 | **798733** |  |
| #32 | 'heart arrest'/de | **59987** |  |
| #31 | 'sudden cardiac death'/de | **9184** |  |
| #30 | 'out of hospital cardiac arrest'/de | **5848** |  |
| #29 | 'experimental heart arrest'/de | **12** |  |
| #28 | 'cardiopulmonary arrest'/de | **4065** |  |
| #27 | 'diabetes mellitus'/de | **479019** |  |
| #26 | 'wolfram syndrome'/de | **748** |  |
| #25 | 'pregnancy diabetes mellitus'/de | **24647** |  |
| #24 | 'nonketotic diabetic coma'/de | **1006** |  |
| #23 | | 'non insulin dependent diabetes mellitus'/de | **197805** |
| #22 | 'newborn diabetes mellitus'/de | **298** |  |
| #21 | 'maternally inherited diabetes and deafness'/de | **182** |  |
| #20 | 'lipoatrophic diabetes mellitus'/de | **178** |  |
| #19 | 'latent autoimmune diabetes in adults'/de | **103** |  |
| #18 | 'insulin dependent diabetes mellitus'/de | **98932** |  |
| #17 | 'impaired glucose tolerance'/de | **24668** |  |
| #16 | 'streptozotocin-induced diabetes mellitus'/de | **17129** |  |
| #15 | 'experimental diabetic nephropathy'/de | **79** |  |
| #14 | 'alloxan-induced diabetes mellitus'/de | **3809** |  |
| #13 | 'experimental diabetes mellitus'/de | **8843** |  |
| #12 | 'diabetic stomach paresis'/de | **240** |  |
| #11 | 'diabetic retinopathy'/de | **34612** |  |
| #10 | 'diabetic obesity'/de | **2565** |  |
| #9 | 'diabetic neuropathy'/de | **20934** |  |
| #8 | 'diabetic nephropathy'/de | **35126** |  |
| #7 | 'diabetic macular edema'/de | **3875** |  |
| #6 | 'diabetic ketoacidosis'/de | **10074** |  |
| #5 | 'diabetic hypertension'/de | **717** |  |
| #4 | 'diabetic foot'/de | **11857** |  |
| #3 | 'diabetic coma'/de | **2234** |  |
| #2 | 'diabetic cardiomyopathy'/de | **2506** |  |
| #1 | 'diabetic angiopathy'/de | **9597** |  |

Embase Session Results (Jul 5, 2018)

Database: Ovid MEDLINE(R) ALL <1946 to July 03, 2018>

Search Strategy:

--------------------------------------------------------------------------------

1 diabetes mellitus.mp. or exp Diabetes Mellitus/ (445430)

2 diabetes.mp. (545858)

3 diabetic.mp. (236780)

4 diabetes complications.mp. or Diabetes Complications/ (41657)

5 out of hospital cardiac arrest.mp. or exp Out-of-Hospital Cardiac Arrest/ (5397)

6 heart arrest.mp. or exp Heart Arrest/ (49833)

7 asystole.mp. (3405)

8 asystoly.mp. (18)

9 asystolia.mp. (138)

10 circulation arrest.mp. (97)

11 heart asystole.mp. (4)

12 heart standstill.mp. (8)

13 circulatory arrest.mp. (5537)

14 1 or 2 or 3 or 4 (610243)

15 5 or 6 or 7 or 8 or 9 or 10 or 11 or 12 or 13 (56191)

16 14 and 15 (1006)

***************************

**Medline search strategy**

**Appendix S1: Embase and Medline Search strategy**
